# Effect of driving pressure on early postoperative lung gas distribution in supratentorial craniotomy: a randomized controlled trial

**DOI:** 10.1186/s12871-023-02144-7

**Published:** 2023-05-22

**Authors:** Feifei Liu, Wei Zhang, Zhanqi Zhao, Xin Xu, Minyu Jian, Ruquan Han

**Affiliations:** 1 Department of Anesthesiology, Beijing Tiantan Hospital, Capital Medical University, No. 119, Southwest 4th Ring Road, Fengtai District, Beijing, 100070 China; 2 Department of Anesthesiology, Beijing Fangshan Liangxiang Hospital, Beijing, China; 3 Institute of Technical Medicine, Furtwangen University, Villingen-Schwenningen, Germany

**Keywords:** Supratentorial craniotomy, Postoperative pulmonary complications, Global inhomogeneity index, Driving pressure, Electrical impedance

## Abstract

**Background:**

Neurosurgical patients represent a high-risk population for postoperative pulmonary complications (PPCs). A lower intraoperative driving pressure (DP) is related to a reduction in postoperative pulmonary complications. We hypothesized that driving pressure-guided ventilation during supratentorial craniotomy might lead to a more homogeneous gas distribution in the lung postoperatively.

**Methods:**

This was a randomized trial conducted between June 2020 and July 2021 at Beijing Tiantan Hospital. Fifty-three patients undergoing supratentorial craniotomy were randomly divided into the titration group or control group at a ratio of 1 to 1. The control group received 5 cmH_2_O PEEP, and the titration group received individualized PEEP targeting the lowest DP. The primary outcome was the global inhomogeneity index (GI) immediately after extubation obtained by electrical impedance tomography (EIT). The secondary outcomes were lung ultrasonography scores (LUSs), respiratory system compliance, the ratio of the partial pressure of arterial oxygen to the fraction of inspired oxygen (PaO_2_/FiO_2_) and PPCs within 3 days postoperatively.

**Results:**

Fifty-one patients were included in the analysis. The median (IQR [range]) DP in the titration group versus the control group was 10 (9–12 [7–13]) cmH_2_O vs. 11 (10–12 [7–13]) cmH_2_O, respectively (*P* = 0.040). The GI tract did not differ between groups immediately after extubation (*P* = 0.080). The LUS_S_ was significantly lower in the titration group than in the control group immediately after tracheal extubation (1 [0–3] vs. 3 [1–6], *P* = 0.045). The compliance in the titration group was higher than that in the control group at 1 h after intubation (48 [42–54] vs. 41 [37–46] ml·cmH_2_O^-1^, *P* = 0.011) and at the end of surgery (46 [42–51] vs. 41 [37–44] ml·cmH_2_O^-1^, *P* = 0.029). The PaO_2_/FiO_2_ ratio was not significantly different between groups in terms of the ventilation protocol (*P* = 0.117). At the 3-day follow-up, no postoperative pulmonary complications occurred in either group.

**Conclusions:**

Driving pressure-guided ventilation during supratentorial craniotomy did not contribute to postoperative homogeneous aeration, but it may lead to improved respiratory compliance and lower lung ultrasonography scores.

**Clinical trial registration:**

ClinicalTrials.gov NCT04421976.

**Supplementary Information:**

The online version contains supplementary material available at 10.1186/s12871-023-02144-7.

## Introduction

In neurosurgery, due to the long-term use of general anesthesia and postoperative bed rest, the risk of postoperative atelectasis and pulmonary infection is increased [1]. Brain injury contributes to an inflammatory environment, which makes the lung tissue more vulnerable to mechanical ventilation, surgery and other factors [2]. The incidence of postoperative pulmonary complications (PPCs) in neurosurgery is 4-25% [3–5]. The key to anesthesia management in supratentorial craniotomy is to avoid hypoxemia, poor cerebral perfusion and increased intracranial pressure (ICP). However, the commonly used protective ventilation strategy, which includes a recruitment maneuver and a higher positive end-expiratory pressure (PEEP), increases ICP and reduces cerebral perfusion in patients undergoing craniotomy [1, 6]. Therefore, it is clinically important to determine how to balance the benefits and harms of mechanical ventilation to the lung and brain at the same time.

Optimization of the ventilation strategy can minimize iatrogenic injury in previously healthy lungs, reducing the incidence of PPCs [7]. More recent studies have suggested driving pressure (DP) as a viable target for lung-protective ventilation [7, 8]. DP is an interesting physiologic variable that has been associated with lung complications retrospectively in patients with lung injury or acute respiratory distress syndrome (ARDS) [9, 10]. A meta-analysis included data from 17 randomized controlled trials, including 2250 patients, and compared low with high PEEP during ventilation with different tidal volumes (TVs). The study found that the setting of TV and PEEP aimed at reducing DP in mechanical ventilation can reduce PPCs [10]. Furthermore, even in patients with healthy lungs, it is assumed that high DP is associated with increased morbidity [10, 11]. Therefore, “lowest DP”-based ventilation has been proposed as a new direction. However, no trials have evaluated the role of DP in early pulmonary ventilation distribution after supratentorial craniotomy.

Electrical impedance tomography (EIT) has been used as a noninvasive, radiation-free, bedside technique for assessing the regional distribution of pulmonary ventilation and perfusion and has a good correlation with computed tomography (CT) and X-ray [12]. Lung ultrasonography (LUS) has been used in adults to evaluate lung aeration and oxygenation and to detect atelectasis caused by anesthesia, with higher LUS scores (LUSs) indicating worse lung aeration [13, 14].

Considering the feasibility and usefulness of these methods, we investigated whether the lowest DP could contribute to the postoperative homogeneous aeration using EIT and LUS.

## Materials and methods

### Study design

This was a single-center, randomized, parallel group, patient and outcome assessor-blinded trial exploring a ventilation strategy targeting DP during supratentorial craniotomy conducted between June 21, 2020, and July 1, 2021, at Beijing Tiantan Hospital, Capital Medical University. The study adhered to the Consolidated Standards of Reporting Trials (CONSORT) guidelines. The study was approved by the Ethics Committee of China (ChiECRCT20200137) on June 12, 2020, and registered at ClinicalTrials.gov (NCT04421976). Participants were included after obtaining written informed consent.

### Study population

Participants were recruited if they met the following criteria: Glasgow Coma Scale score of more than 8 points, age between 18 and 70 years, American Society of Anesthesiologists (ASA) level ≥ II, mechanical ventilation duration ≥ 2 h, and elective supratentorial craniotomy. Patients were excluded if they met at least one of the following criteria: preexisting severe respiratory disease (chronic lung disease, pneumonia, acute lung injury or acute respiratory distress syndrome) or heart disease, dysphagia resulting from preoperative cranial nerve damage, body mass index (BMI) ≥ 35 kg ⋅ m^− 2^, mechanical ventilation > 1 h within 2 weeks before the operation, progressive neuromuscular disease, pregnancy and any contraindication to EIT or LUS scan.

### Randomization and blinding

Randomization was conducted using computer-generated random numbers sealed in opaque envelopes. Patients were randomly allocated into two groups by the corresponding envelope. Knowing the group task, the anesthesiologist was responsible for the intervention, and the other researchers, blinded to the random allocation, participated in the follow-up visit and data analysis. Chest EIT and lung ultrasonography were performed by the relevant technicians and analyzed by a researcher.

### Anesthesia

Smokers had quit smoking more than four weeks before surgery. For intravenous induction, sufentanil (0.2–0.3 µg ⋅ kg^− 1^), propofol (2–2.5 mg ⋅ kg^− 1^), and rocuronium (0.6 mg ⋅ kg^− 1^) were used. Anesthesia was maintained with sevoflurane (0.4–0.5 MAC), remifentanil (0.05–0.2 µg ⋅ kg^− 1^ ⋅ min^− 1^), propofol (3–4 mg ⋅ kg^− 1^ ⋅ h^− 1^) and rocuronium (0.2 mg ⋅ kg^− 1^, per 50–60 min). Routine perioperative monitoring included invasive blood pressure and the bispectral index (BIS). Mean arterial pressure (MAP) was maintained at > 70 mmHg. Neuromuscular blockade was reversed with neostigmine 0.04 mg·kg-1 and atropine 0.02 mg·kg^− 1^ when the train-of-four (TOF) ratio was below 0.9. Extubation was performed when patients recovered consciousness and demonstrated sufficient spontaneous breathing. All patients were transferred to the postanesthesia care unit (PACU) after successful extubation and monitored for at least 1 h in the PACU. Supplemental oxygen was administered at 3 L·min^− 1^ via a face mask. Postoperative pain was assessed at 20 min postoperatively by using the visual analog scale (VAS).

### Ventilation protocol

Volume-controlled mechanical ventilation was provided (Datex Ohmeda S/5 Advance, General Electric Healthcare, Helsinki, Finland). All patients were preoxygenated with a 0.8 FiO_2_ before tracheal intubation for 3 min. After intubation, the initial settings were TV 8 ml ⋅ kg^-1^ of predicted body weight (PBW), fresh gas 2 L·min^-1^, FiO_2_ 0.4 or higher if the SpO_2_ < 92%, inspiratory to expiratory ratio 1:2, and a respiratory rate (RR) adjusted according to normocapnia (PaCO_2_ between 35 and 45 mmHg). Mechanical ventilation parameters, such as plateau pressure (Pplat), peak pressure (Ppeak) and compliance, were acquired from the anesthetic machine. In the control group, PEEP was set at 5 cmH_2_O until respiratory restoration [15]. In our study, the ventilation strategy with a fixed PEEP of 5 cmH_2_O was applied safely to patients with brain tumors [15]. The retrospective study showed that the application of PEEP had no effect on either ICP or cerebral perfusion pressure (CPP) for those without severe lung injury. We adopted the titration of PEEP strategy similar to the literature [16]. In the titration group, the PEEP value was increased stepwise by 1 cmH_2_O from 2 cmH_2_O to 10 cmH_2_O after the patient was placed in position, with TV and RR unchanged. Each level was maintained for 10 breathing cycles, with DP in the last cycle recorded. Then, the level producing the lowest DP was identified as “optimal”, and the individual PEEP was maintained throughout mechanical ventilation. The Pplat should not exceed 30 cmH_2_O in each group; otherwise, the titration would be terminated in advance.

### Measurements

The dynamic changes in aeration distribution can be visualized and evaluated by EIT [17]. The global inhomogeneity index (GI) is a measure that describes the regional ventilation distribution and homogeneity [18]. A lower GI implies more ventilation homogeneity in the lung [18, 19]. In the present study, EIT measurements were performed at three specific time points (PulmoVista 500, Draeger Medical, Lübeck, Germany): preinduction, immediately after extubation and 1 h after extubation. An oblique belt with 16 electrodes was placed between the 5th and 6th intercostal spaces, and the data were recorded after the main cable was linked. Data for 5 min at each time point were recorded, and the belt position on the patient’s skin was marked. GI [20] was calculated offline using customized software to evaluate the distribution of ventilation.

LUS can be used as a fast and easily available bedside test to evaluate lung areation. A-lines are a single line or multiple lines parallel to the pleural line and occur in normal lungs [14, 21]. B-lines are defined as hyperechoic vertical artifacts that originate from the pleural line, reach the bottom of the screen without fading and move synchronously with lung sliding [22]. Sets of at least three hyperechoic B-lines arising from the pleural line in one intercostal space are indicative of interstitial lung syndrome [23]. Each hemithorax was divided into six quadrants using two longitudinal lines (anterior and posterior axillary) and one axial line at the level of the nipples. Each quadrant was assigned a score of 0 to 3 according to a modified grading system: 0, normal lung with sliding pleura and A-lines; 1, three or more scattered B-lines; 2, coalescent B-lines; and 3, consolidated lung. The LUSs (0–36) were then calculated by summing the 12 individual quadrant scores, with higher scores indicating more severe aeration loss [24, 25]. A complete ultrasound examination using an ultrasound machine (Sonosite M-Turbo, Sonosite, USA) and a 6–13 MHz linear transducer array (L25) required an average duration of 10 min. The lung ultrasonic measurement time was consistent with the EIT. LUS was performed by trained anesthesiologists (Fei L and Wei Z, with 1 year and 3 years of experience in LUS, respectively).

To evaluate gas exchange, arterial blood gas was tested (ABL 800, Radiometer, Copenhagen, Denmark), and pH and PaO_2_ were recorded.

Brain relaxation was evaluated by a neurosurgeon after craniotomy and before cutting the dura mater [26]. It was assigned a score from 1 to 4 points: scores of 1 and 2 points were considered soft/adequate/no swelling/moderate swelling and good, and scores of 3 and 4 points were considered tight and pronounced swelling and bad.

Pulmonary complications within 3 days after the operation were evaluated by the Melbourne Group Scale Version 2 (MGS-2) [27]. PPCs were diagnosed if four or more factors were present.

### Outcomes

The primary outcome was the GI value immediately after extubation. The secondary outcomes were LUSs, respiratory system compliance, PaO_2_/FiO_2_ ratio and PPCs within 3 days postoperatively.

### Sample size calculation

The sample size was estimated for a previous study. A difference of 0.1 in GI between groups according to a previous study was detected [20], with an alpha level of 0.05 and an SD of 10% using the independent t test at 90% power. Considering a dropout rate of 5%, 24 participants were needed per group.

### Statistical analysis

Categorical variables are reported as the number (proportion) of patients, normally distributed data are presented as the mean and standard deviation (SD), and nonnormally distributed data are presented as the median (IQR [range]). The Kolmogorov–Smirnov test was used to assess the normality of the distribution. For baseline characteristics between groups, the standardized mean difference(SMD) with 95% confidence intervals (95% CIs) was calculated. Two-tailed unpaired Student’s t test was conducted to compare continuous variables between two groups. The Mann–Whitney U test was conducted to assess the differences between groups for nonnormally distributed data (age, BMI, ventilation duration, intraoperative fluid input, intraoperative bleeding and intraoperative urine). The chi-square test or Fisher’s exact test was used to compare two or more proportions (PPC with 3 days). Two-way ANOVA was conducted to evaluate the effects of group, time, and the interaction on GI, LUSs, respiratory mechanics, PaO_2_/FiO_2_ ratio and hemodynamic variables. Because of the repeated measurement, the Holm–Bonferroni method was used to adjust the P value for outcomes. Data analysis was performed using GraphPad Prism 8.0 (GraphPad software, USA). *P* less than 0.05 was considered to indicate a significant difference.

## Results

**Fig. 1:**
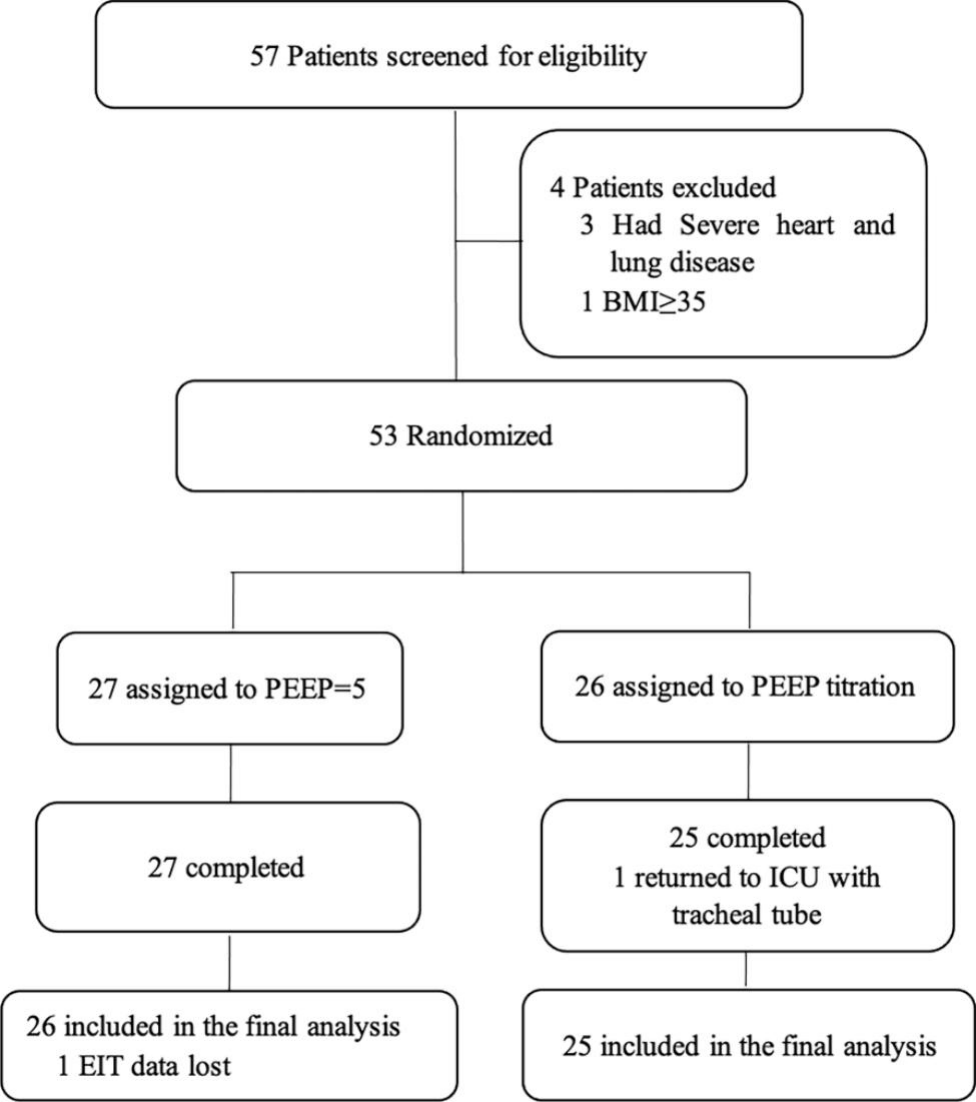
Flow diagram of patients undergoing elective supratentorial craniotomy. BMI, body mass index; PEEP, positive end-expiratory pressure; EIT, electrical impedance tomography; ICU, intensive care unit

Of 57 patients assessed for eligibility, 4 patients did not meet the inclusion criteria, so 53 patients were randomized into two groups and received the intended interventions. One patient was excluded because he returned to the intensive care unit (ICU) with a tracheal tube after the operation in the titration group, and 1 patient was excluded due to technique problems in EIT in the control group. Finally, 51 patients were enrolled in the analyses (Fig. 1). There were two patients with coronary heart disease in the control group but no clinical symptoms. (Table 1).

**Table 1:** Demographics and surgery characteristics

Characteristics	control group (n = 26)	titration group (n = 25)	SMD [95%CI]
Age; yr	48 (8)	47 (9)	0.11 [-0.45,0.67]
Sex; male/female	14/12	12/13	0.12 [-0.43,0.67]
BMI; kg·m^− 2^	25.1 (2.9)	24.6 (3.5)	0.13 [-0.43,0.70]
ASA, II, n (%)	0(0)	0(0)	
III, n (%)	26 (100)	25 (100)	
Hemoglobin, g·dl^− 1^	13.3(1.6)	13.2 (1.4)	0.12 [-0.44,0.68]
Smoking, n (%)	8 (31)	9 (36)	0.11 [-0.44,0.66]
Comorbidities, n (%)			0.51 [-0.05,1.06]
Hypertension	7 (27)	5 (20)	
Diabetes	3 (12)	2 (8)	
Heart disease	2 (8)	0 (0)	
Position, n (%)			0.13 [-0.42,0.68]
Supine	23 (88)	21 (84)	
Lateral	3 (12)	4 (16)	
Type of tumor, n (%)			0.46 [-0.10,1.02]
Glioma	13 (50)	12 (48)	
Meningioma	7 (27)	10 (40)	
Craniopharyngioma	1 (4)	1 (4)	
Pituitary tumor	1 (4)	0 (0)	
Metastatic tumor	1 (4)	1 (4)	
Others	3 (11)	1 (4)	

Data are presented as mean(standard deviation) or a frequency with proportion(%). The standardized mean difference(SMD) with 95% confidence interval (95% CI) is calculated for the data. BMI body mass index, ASA American Society of Anesthesiologists

DP in the titration group was significantly lower than in the control group, with a median (IQR [range]) of 10 (9–12 [7–13]) cmH_2_O vs. 11 (10–12 [7–13]) cmH_2_O (*P* = 0.040), respectively, corresponding to an optimal PEEP level of 3 (2–4 [2–7]) cmH_2_O in the titration group.

**Fig. 2:**
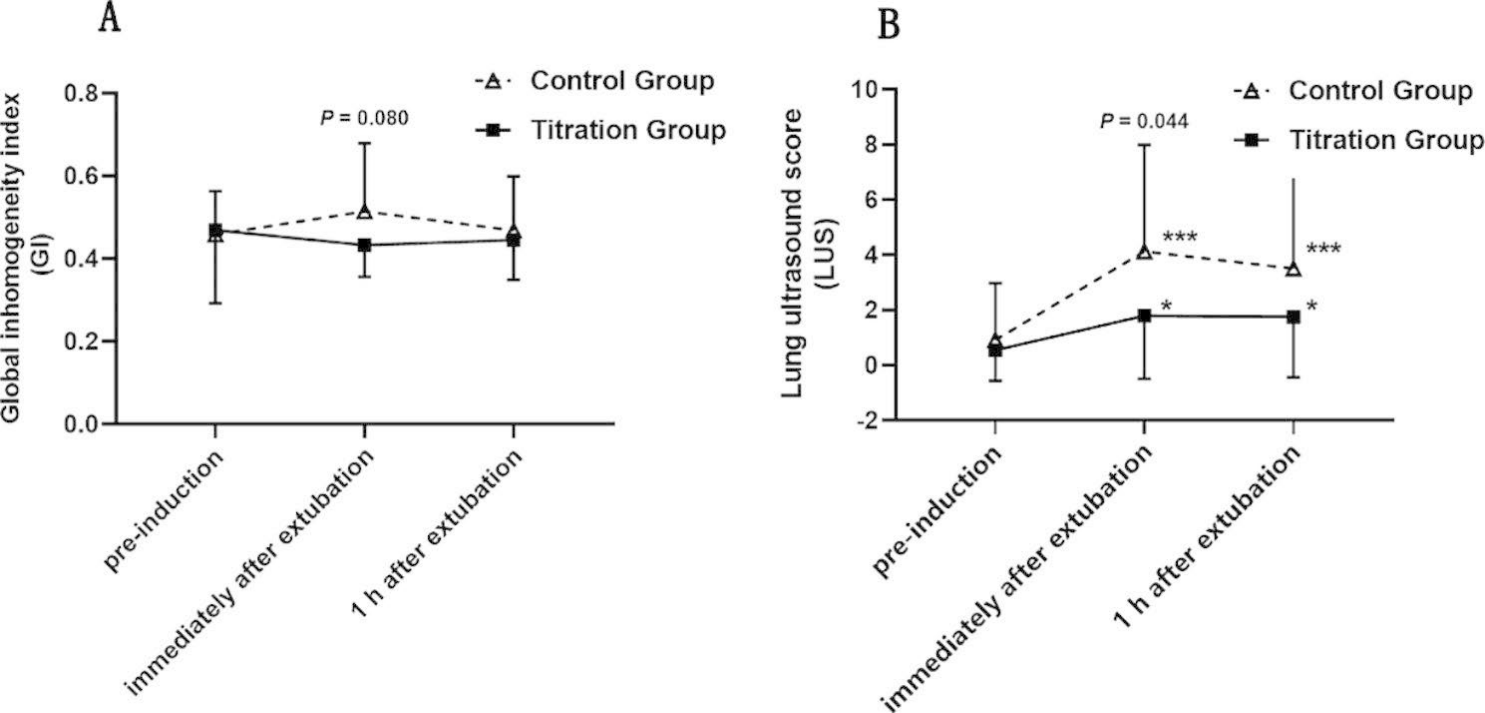
Global inhomogeneity index obtained from electrical impedance tomography and lung ultrasonography scores at different time points Captions: *, p < 0.05; ***, p < 0.001 compared to baseline

The GI in the titration group showed a tendency to fall immediately after extubation compared with baseline but did not differ between different time points. The differences between the two groups were not significant (*P* = 0.080) immediately after extubation. The GI returned to baseline 1 h after tracheal intubation in both groups (Fig. 2A).

**Fig. 3:**
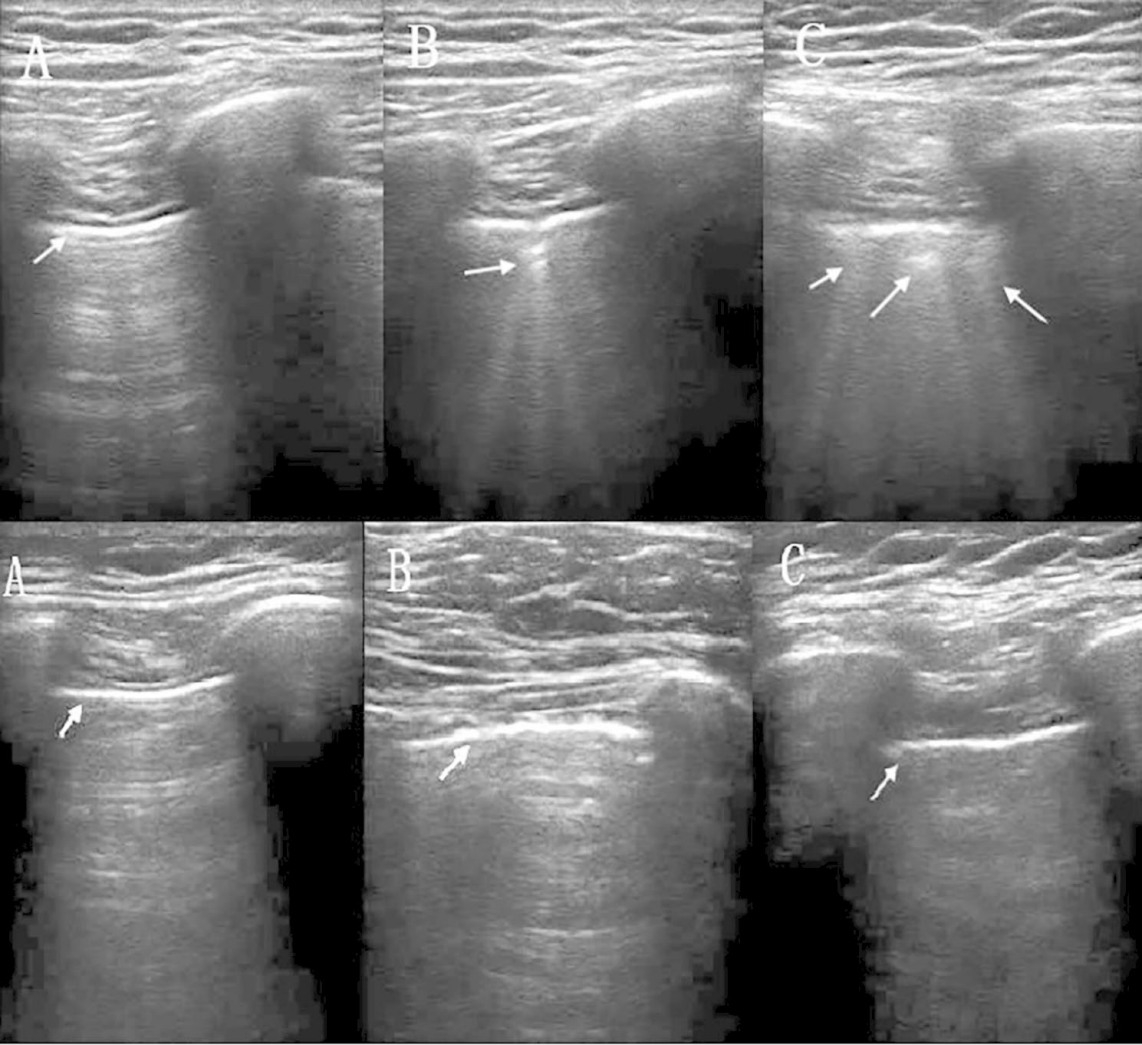
Representative lung ultrasonography images at different time points The first group is the control group, and the second group is the titration group. **(A)** Preinduction. **(B)** Immediately after extubation. **(C)** One hour after extubation. At least three hyperechoic B-lines can be seen originating from the pleural line in the control group either immediately or 1 h after extubation

In both groups, there was an increase in the LUSs immediately after extubation and 1 h after extubation (*P* < 0.001 for the control group and *P* < 0.05 for the titration group), implying a significant loss of aeration in the lung after supratentorial craniotomy. Furthermore, the values were obviously higher in the control group than in the titration group immediately after extubation (1 [0–3] vs. 3 [1–6], *P* = 0.045), suggesting that DP-titrated ventilation prevents aeration loss (Fig. 2B). Representative lung ultrasonography images at different time points are shown in Fig. 3.

The titration group showed better respiratory system parameters than the control group. The compliance in the titration group was higher than that in the control group at 1 h after intubation and at the end of surgery (*P* = 0.011 vs. *P* = 0.029). The Pplat in the titration group was lower than that in the control group (*P* = 0.003). However, the Ppeak was not different between groups, and these assessment levels were within the safe limit (Table 2).

The changes in the PaO_2_/FiO_2_ ratio were lower in both groups 1 h after intubation, immediately after extubation and 1 h after extubation than at preinduction. PaO_2_/FiO_2_ in the titration group and in the control group at 1 h after intubation were 390 [330–428] vs. 321 [278–377]), as well as immediately after extubation (373 [338–409] vs. 323 [278–377]). However, there was no significant difference between groups in terms of the ventilation protocol (*P* = 0.117) (Table 2).

**Table 2:** Patient ventilatory mechanics and gas analysis

Characteristics	control group (n = 26)	titration group	p		
		(n = 25)	Time	Group	Interaction
Compliance (ml·cmH_2_O^− 1^)			0.082	0.045	< 0.001
immediately after intubation	46 (42,49)	45 (42,48)			
1 h after intubation	41 (37,46)	48 (42,54) *			
end of surgery	41 (37,44)	46 (42,51) *			
Ppeak (cmH_2_O)			< 0.001	0.065	0.262
immediately after intubation	14 (13,16)	14 (12,16)			
1 h after intubation	18 (17,19)	15 (14,18)			
end of surgery	18 (17,19)	16 (15,19)			
Pplat (cmH_2_O)			< 0.001	0.064	0.003
immediately after intubation	14 (13,15)	13 (12,15)			
1 h after intubation	17 (14,18)	15 (13,17)			
end of surgery	17 (16,18)	15 (14,17)			
PaCO_2_			< 0.001	0.648	0.042
preinduction	38 (36,41)	37 (35,39)			
1 h after intubation	36 (35,39)	35 (33,37)			
immediately after extubation	47 (42,50)	46 (41,49)			
1 h after extubation	39.6 (5.4)	43 (38,47)			
pH			< 0.001	0.612	0.971
preinduction	7.42 (7.41,7.44)	7.43 (7.40,7.46)			
1 h after intubation	7.43 (7.41,7.44)	7.42 (7.40,7.46)			
immediately after extubation	7.34 (7.32,7.38)	7.35 (7.33,7.40)			
1 h after extubation	7.37 (7.35,7.40)	7.37 (7.36,7.40)			
PaO_2_/FiO_2_			0.001	0.011	0.117
preinduction	386 (341,473)	414 (384,475)			
1 h after intubation	321 (274,382)	390 (330,428) *			
immediately after extubation	323 (278,377)	373 (338,409) *			
1 h after extubation	309 (241,361)	382 (296,494)			

Data are presented as the Median (IQR).Ppeak, airway peak pressure; Pplat, airway plateau pressure. PaCO_2_, partial pressure of carbon dioxide in arterial blood; PaO_2_/FiO_2_, the ratio of arterial partial pressure of oxygen to inspiratory oxygen fraction. *, p < 0.05 compared to the control group

At the 3-day follow-up, no PPCs, defined as those with an MGS-2 of at least 4, occurred in either group. During the entire surgical period, the number of patients using vasopressors and requiring crystalloid fluid infusion did not differ between groups(Table 3).

**Table 3:** Surgery and postoperative characteristics

Characteristics	control group (n = 26)	titration group (n = 25)	p
Tidal volume; ml	450 (420,550)	460 (430,530)	0.96
RR; breaths·min^− 1^	12 (12,12)	12 (12,13)	0.745
Ventilation duration; min	306 (230,336)	290 (215,327)	0.55
Intraoperative fluid input; ml	2482 (2000,2500)	2500 (2000,2500)	0.43
Intraoperative bleeding; ml	1200 (1000,1800)	1200 (1000,1825)	0.789
Intraoperative urine; ml	1400 (1200,1950)	1400 (1150,1950)	0.906
Brain relaxation, n (%)			0.459
1 point	3 (12)	2 (8)	
2 points	11 (42)	14 (56)	
3 points	10 (38)	9 (36)	
4 points	2 (8)	0 (0)	
Vasoactive drugs, n (%)	2 (8) 0 (0)	7 (28)	0.075
VAS score > 3, n (%)		0 (0)	> 0.99
PPCs; Yes	0 (0)	0 (0)	> 0.99

Data are presented as the Median (IQR) or a frequency with proportion(%). RR, respiratory rate; VAS, visual analog scale; PPCs, postoperative pulmonary complications

## Discussion

The main findings of this study included the following: (1) compared with a fixed 5 cmH_2_O PEEP, driving pressure-targeted PEEP could not contribute to a more homogeneous distribution, but it led to less aeration loss and improved respiratory compliance for patients who underwent supratentorial craniotomy, and (2) the level of PEEP required for optimal ventilation for neurosurgery was found to be lower than that currently used in clinical practice. Thus, these findings strongly suggest that driving pressure-targeted PEEP can be used in neurosurgery. Protective lung ventilation has seldom been used in neurosurgery in previous studies because low TV and PEEP are considered to increase ICP in patients with intracranial masses [15]. Recently, a small randomized clinical trial of patients who underwent elective neurosurgery showed that ICP did not differ between patients allocated to traditional and protective ventilation, and dural tension was acceptable for surgery in all patients [28]. Our findings are in line with those of that study, which showed that intracranial pressure and dural tension were not significantly increased. Furthermore, the number of patients requiring vasoactive drugs and crystalloid fluid infusion did not differ significantly between groups. Therefore, this ventilation strategy is relatively safe.

DP is a significant mediator of PPCs. A DP of greater than 16 cmH_2_O has been associated with an increased risk of PPCs for ARDS and elective cardiac surgeries [9, 29]. An international consensus on lung protection has also recommended avoiding an increase in DP [30]. In our single center, the DP was 9 to 12 cmH_2_O (median, 10) in the titration group, which was lower than the median of 11 (10–12) in the control group. The optimal PEEP values ranged between 2 and 7 cmH_2_O with a median of 3 cmH_2_O. DP is the difference between Pplat and PEEP [9]. A previous study presented an increased PEEP to optimize (minimize) driving pressure [9]. However, our study had different outcomes. We demonstrated that optimal PEEP, compared with the fixed PEEP of 5 cmH_2_O, improved compliance intraoperatively and decreased Pplat. The higher compliance and lower DP obtained strongly suggested minimizing lung functional overdistention and collapse. DP scales the tidal volume dependence of the functional lung size and PEEP, so individualizing the ventilatory settings intraoperatively and achieving an optimum DP adapted to the functional lung size is important [31]. This study supported the idea that a fixed PEEP is not suitable due to the individual characteristics of the patient and surgery [16, 32, 33]. Driving pressure-targeted PEEP provides the optimum compromise to improve aeration. In a previous study in thoracic surgery, the incidence of PPCs was 12.2% with conventional protective ventilation and 5.5% with driving pressure-guided ventilation, although the difference in DP between the two groups was only 1 cm H_2_O [16]. However, our study was unable to test differences in clinical outcomes, and pulmonary complications within 3 days after surgery were rare in both groups. More studies are needed to confirm the reduction in pulmonary complications 3 days after the operation. In the present study, each PEEP maintained 10 breathing cycles, which was adopted from a previous study [16]. Several studies have suggested that a new balance of mechanics and imaging could be achieved within a minute [33–35]. Nevertheless, 10 breaths might not be sufficient in some patients, which might lead to an underestimated optimal PEEP.

The GI directly represents global inhomogeneity in tidal ventilation [18, 36], which varies depending on the physiologic state of the lungs. Although the GI in the titration group showed a tendency to fall below baseline immediately after extubation, no significant differences were found either between groups or with baseline values in this study (Fig. 2A). The results are in line with a trial in which no statistically significant change was found in GI values after different levels of PEEP on EIT in the lateral decubitus position during elective urologic surgery [37]. That study demonstrated that compliance with the lateral position was not accurate and was not correlated with the regional distribution of ventilation. Individualized high PEEP titrated using EIT prevented atelectasis in obese patients during anesthesia but not the early postoperative period [38]. They observed that the differences in ventilation distribution during mechanical ventilation vanished after extubation. These findings might explain why the GI is related to the type of surgery and position. Further study will be needed to test its effects on GI.

In this study, the LUSs were higher in the control group than in the titration group immediately and 1 h after extubation (Fig. 2B). This revealed that the lung-protective strategy of optimal PEEP could compensate for lung aeration deterioration. It exerts its effects on less aeration loss during the whole operation and 1 h after the extubation stage. The LUSs of the titrated PEEP group were significantly lower than those of the constant PEEP group and the conventional ventilation group in elderly patients undergoing laparoscopic surgery [39]. Immediately after extubation, two patients exhibited loss of ventilation corresponding to coalescent B-lines in the control group. Immediately after extubation, two patients exhibited loss of ventilation corresponding to coalescent B-lines in the control group. The presence of B-line and hypoechoic juxta-pleural consolidations using air bronchograms or a tissue-like pattern are most helpful to check perioperative atelectasis [22, 40]. However, the patients were not checked for a thoracic CT. The incidence of atelectasis was not detected in our study.

Individualized PEEP improved respiratory system mechanics, with higher respiratory system compliance and lower Pplat (Table 2). Perioperative PaO_2_/FiO_2_ was not significantly different between the ventilation protocol groups (*P* = 0.117) (Table 2). Postoperative PaO_2_/FiO_2_ might be a potential target independently associated with PPCs and mortality [41, 42]. No hypoxemia was observed in either group. In patients who underwent open abdominal surgery in a previous study, the changes in the LUSs were moderately correlated with changes in PaO_2_/FiO_2_ [23].

A recruitment maneuver (RM) might be used prior to PEEP titration to reopen collapsed lung regions. However, several studies have indicated that its adverse effects cannot be ignored. Nemer et al. [43] observed that RM led to a significant increase in ICP and a significant decrease in MAP and CPP, with no improvement in oxygenation. The results were consistent with that study, which found that a single RM before the induction of pneumoperitoneum could not improve respiratory mechanics and oxygenation in elderly patients who underwent robotic-assisted radical prostatectomy [44]. Currently, RM is seldom applied in neurosurgical patients. The “optimal” PEEP might be different when noncorrected lung collapse is presented, which was not explored in the current study.”

There were several limitations in our study. First, the sample size was small, but it was adequate for achieving significant differences in the endpoints between groups. Second, because difficulties with ventilation and intubation are unpredictable, the need for preoxygenation is desirable in all patients. All patients were preoxygenated with a 0.8 FiO_2_ before tracheal intubation for 3 min in our study. A previous study indicated that preoxygenation with a 0.6 or 0.8 FiO_2,_ which causes less atelectasis than preoxygenation with 100% oxygen [45]. An FiO_2_ of 0.3 during the induction of anesthesia is not associated with atelectasis [46]. However, lower oxygen concentrations during the induction of anesthesia were not given. Third, the incremental PEEP titration was a limitation in our study design. An incremental PEEP trial results in variable end-inspiratory recruitment, which affects end-expiratory recruitment at any particular PEEP level. The individualized recruitment maneuver and decremental PEEP titration for a further reduction in DP and a more homogeneous distribution of lung gas did not support our study design. The safety of increment titration was not evaluated by cerebral hemodynamics through transcranial Doppler (TCD) or near-infrared spectroscopy (NIRS). Fourth, we did not evaluate the relationship between the degrees of brain tumors and PPCs. Due to different degrees of severity of brain tumors, the physical condition of patients varies. Fifth, we did not perform EIT and lung ultrasonography at the time of the surgical procedure or 3 days after the operation because of clinical restrictions. Finally, future studies should involve larger populations to assess the impact of individualized PEEPs. Further studies are also needed to explore the optimal ventilation strategy, in which LUSs will be used to estimate atelectasis 24 h after surgery.

## Conclusions

Driving pressure-guided ventilation during supratentorial craniotomy did not contribute to postoperative homogeneous aeration, but it may lead to improved respiratory compliance and lower lung ultrasonography scores.

## Electronic supplementary material

Below is the link to the electronic supplementary material.

Supplementary Material 1

Supplementary Material 2

## Data Availability

The datasets used and analysed during the current study available from the corresponding author on reasonable request.

## Abbreviations

PPCs: Postoperative pulmonary complications
DP: Driving pressure
EIT: Electrical impedance tomography
GI: Global inhomogeneity
LUSs: Lung ultrasonography scores
ICP: Intracranial pressure
PEEP: Positive end-expiratory pressure
PaO_2_/FiO_2_: The partial pressure of arterial oxygen to the fraction of inspired oxygen
ARDS: Acute respiratory distress syndrome
TV: Tidal volume
CT: Computed tomography
LUS: Lung ultrasonography
ASA: American Society of Anesthesiologists
BMI: Body mass index
BIS: Bispectral index
MAP: Mean arterial pressure
TOF: Train-of-four
PACU: Post-anesthesia care unit
VAS: Visual analog score
Pplat: Plateau pressure
Ppeak: Peak pressure
CPP: Cerebral perfusion pressure
ICU: Intensive care unit
RM: Recruitment maneuver
TCD: Transcranial doppler
NIRS: Near-infrared spectroscopy
