# Supplementary material for: Effect of driving pressure on early postoperative lung gas distribution in supratentorial craniotomy: a randomized controlled trial
Source: BMC Anesthesiol. 2023 May 22;23:176. doi: 10.1186/s12871-023-02144-7 (PMC10201743; doi:10.1186/s12871-023-02144-7)
Supplement: Supplementary file 1 — Supplementary Material 1 [file 12871_2023_2144_MOESM1_ESM.pdf]

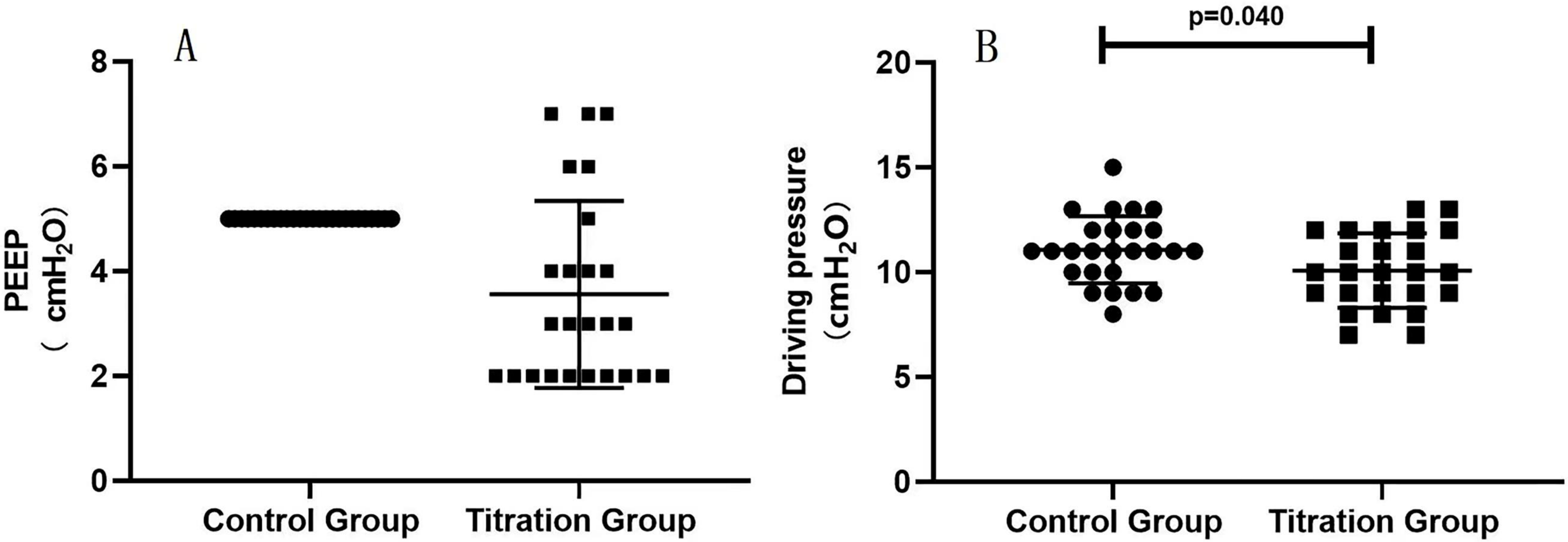


**Supplemental Figure 1** Distribution plot for PEEP level (A) and driving pressure (B). Captions: Significant differences occurred between groups for driving pressure. Lines indicate the median with IQR. PEEP, positive end-expiratory pressure.
