# Supplementary material for: Effect of driving pressure on early postoperative lung gas distribution in supratentorial craniotomy: a randomized controlled trial
Source: BMC Anesthesiol. 2023 May 22;23:176. doi: 10.1186/s12871-023-02144-7 (PMC10201743; doi:10.1186/s12871-023-02144-7)
Supplement: Supplementary file 2 — Supplementary Material 2 [file 12871_2023_2144_MOESM2_ESM.pdf]

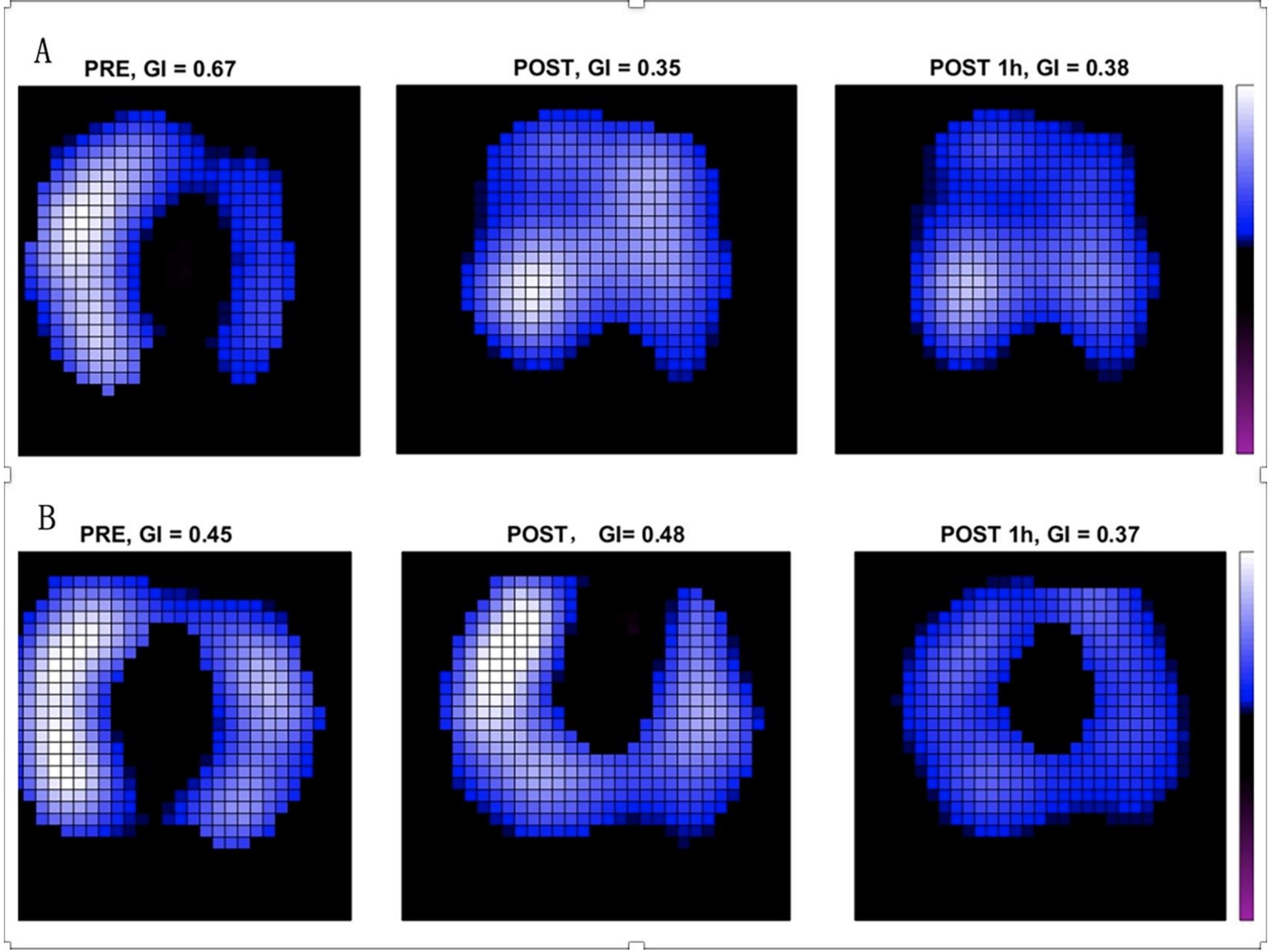

Supplemental Figure 2 Representative EIT images at different time points. Captions: Images A, titration group. Images B, control group. The GI value was obviously lower in the titration group than in the control group immediately after extubation. PRE, preinduction; POST, immediately after extubation; POST 1 h, 1 hour after extubation.
